# RNA Interference-Induced Innate Immunity, Off-Target Effect, or Immune Adjuvant?

**DOI:** 10.3389/fimmu.2017.00331

**Published:** 2017-03-23

**Authors:** Zhongji Meng, Mengji Lu

**Affiliations:** ^1^Department of Infectious Diseases, Taihe Hospital, Hubei University of Medicine, Shiyan, China; ^2^Institute of Virology, University Hospital of Essen, University of Duisburg-Essen, Essen, Germany

**Keywords:** RNA interference, small interfering RNA, innate immunity, immunostimulatory RNA, immunostimulatory small interfering RNAs

## Abstract

RNA interference (RNAi) is a natural cellular mechanism that inhibits gene expression in a sequence-specific manner. In the last decade, RNAi has become a cornerstone in basic biological systems research and drug development efforts. The RNAi-based manipulation of mammalian cells facilitates target identification and validation; assists in identifying human disease etiologies; and expedites the development of treatments for infectious diseases, cancer, and other conditions. Several RNAi-based approaches are currently undergoing assessment in phase I and II clinical trials. However, RNAi-associated immune stimulation might act as a hurdle to safe and effective RNAi, particularly in clinical applications. The induction of innate immunity may originate from small interfering RNA (siRNA) sequence-dependent delivery vehicles and even the RNAi process itself. However, in the case of antagonistic cancers and viral infection, immune activation is beneficial; thus, immunostimulatory small interfering RNAs were designed to create bifunctional small molecules with RNAi and immunostimulatory activities. This review summarizes the research studies of RNAi-associated immune stimulation and the approaches for manipulating immunostimulatory activities.

## Introduction

RNA interference (RNAi) is a post-transcriptional gene regulation mechanism by which small interfering RNAs (siRNAs) induce the sequence-specific degradation of homologous messenger RNA (mRNA) (1). In the past 16 years, RNAi has been widely used in basic biological research and drug development processes. In particular, significant progress has been achieved in the fields of cancer (2), leukemia (3), hepatitis B virus (HBV), and human immunodeficiency virus infections (4). There are currently 45 registered studies.^1^ Clinical trials using siRNA-based drugs have shown efficacy in the treatment of HBV infection (5), respiratory syncytial virus infection (6), and cancer.

The data from a phase IIa clinical study presented at the AASLD Liver Meeting in 2015 demonstrated that ARC-520, a targeted RNAi therapeutic against HBV, effectively reduced HBV surface antigen levels up to a maximum mean 1.5 logs (96.8%) and 1.9 logs (99%) in treatment-naïve patients who tested positive for HBV e-antigen. The direct sustained antiviral effect of a single dose lasted 57 days. ARC-520 has been examined in multiple studies aimed at producing a functional cure for HBV infection (7). Unfortunately, the clinical trials with ARC-520 have been recently stopped by FDA due to the occurrence of sever adverse effect.

In siRNA-based technologies, many studies have reported innate immune stimulation by siRNA and/or the siRNA delivery vehicle (8–11). Various features of the siRNA structure, sequence, and delivery mode have contributed to the immune stimulation effect, leading to undesired effects and the misinterpretation of experimental results, i.e., immunological off-target effects (12). Sledz et al. observed a twofold induction of 52 of the 850 putative interferon (IFN)-stimulated genes (ISGs) upon the use of synthetic siRNAs (13). Bridge et al. reported that the use of DNA vectors that encoded small hairpin RNAs upregulated 27 ISGs. Of note, these authors observed that oligoadenylate synthase-1 (OAS1) was upregulated by 50-fold using one siRNA vector and 500-fold using two vectors (14). However, neither the silencing vectors nor the synthetic siRNAs was effective, suggesting that some aspect of both the siRNA sequence and the delivery method is required to upregulate IFN. Most of the published data of the immune-related side effects of various reagents observed in *in vitro* and *in vivo* experiments that may be helpful in the development and use of microRNA (miRNA)- and RNAi-based methods are stored in the RNAimmuno database^2^ (15).

In a previous study in primary woodchuck hepatocytes (PWHs) with woodchuck hepatitis virus (WHV) infection, we observed that RNAi-mediated WHV suppression upregulated the expressions of Myxovirus resistance A (MxA) and major histocompatibility complex I (MHC-I) genes (16), while further studies revealed that the RNAi process enhances innate immune responses *via* multiple signaling pathways in primary hepatocytes (17).

## siRNA-Associated Immune Stimulation

### Molecular Foundation of Single-Stranded RNA (ssRNA) And Double-Stranded RNA (dsRNA) Immune Sensing

**Figure 1:**
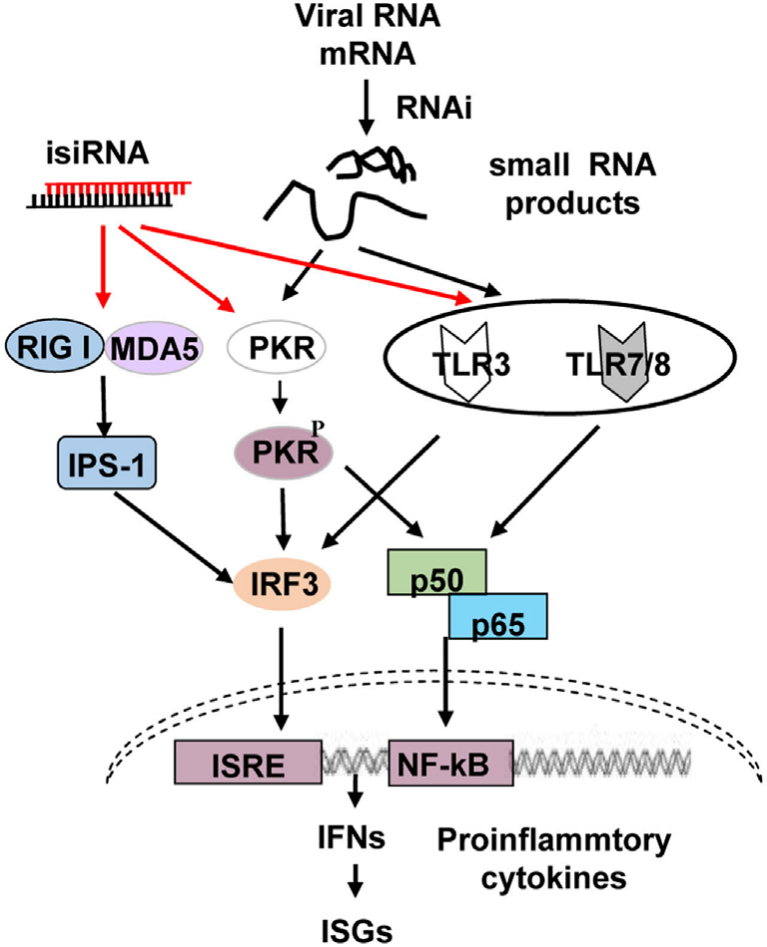
**Schematic representation of RNA interference (RNAi)-associated immunostimulation**. The retinoic acid-inducible gene-I (RIG-I)/melanoma differentiation-associated protein 5 (MAD5), toll-like receptor (TLR) 3, TLR7/8, and PKR signaling pathways can recognize isiRNA; on the other hand, the RNAi process generates small RNAs that may activate PKR, TLR3, and TLR7/8, inducing type I interferon (IFN) and pro-inflammatory cytokine production. isiRNA, immunostimulatory siRNA.

Four or more signaling pathways recognize RNA molecules and induce the production of type I IFN and pro-inflammatory cytokines, including the retinoic acid-inducible gene-I (RIG-I)/melanoma differentiation-associated protein 5 (MDA5), toll-like receptor (TLR) 3, TLR7/8, and dsRNA-dependent protein kinase (PKR) pathways (Figure 1). MDA5 recognizes long RNA molecules, while RIG-I detects the emerging 5′-triphosphate moiety of viral transcripts or genomes of negative-sense ssRNA viruses (18), dsRNAs that are 300–1,000 bp long (19), short blunt-ended dsRNA (siRNA ~21 nucleotides long) (19), and RNase L-generated small self-RNAs (20). Thus, the RIG-I/MDA5 pathway recognizes ssRNA, dsRNAs, siRNAs, and small self-RNAs; TLR3 recognizes dsRNA; and TLR7 and TLR8 identify GU-rich short ssRNA as well as small manmade molecules such as nucleoside analogs and midazoquinolines. These ligands bind to TLR3, and TLR7/8 subsequently activates downstream signaling molecules, including nuclear transcription factor (NF)-κB, IFN regulatory factor (IRF) 1/3/7, c-Jun N-terminal protein kinase, and mitogen-activated protein kinase, activating type I IFNs, chemokines, pro-inflammatory cytokines, antibodies, adhesion molecules, MHCs, and costimulatory molecules (21). PKR was first identified as a sensor that functions in a non-sequence-specific fashion with long (>33 bp) dsRNA sequences, causing activation. Some cellular and viral RNAs containing multiple shorter dsRNA sections and non-Watson–Crick structures can also regulate PKR (22, 23).

### Single- and Double-Stranded siRNA-Induced Sequence-Dependent Inflammatory Cytokine and IFN Activations

Sioud demonstrated that single- and double-stranded siRNAs (sense or antisense strands) could induce interleukin-6 (IL-6) and tumor necrosis factor-α (TNF-α) in adherent peripheral blood mononuclear cells (PBMCs). The fact that only certain sequences induced inflammatory responses suggested that the siRNA-associated immunostimulatory effects were sequence-dependent. TNF-α, IL-6, and IFN-α inductions were chloroquine-sensitive and likely dependent on endosomal TLR signaling, specifically TLR8 (24). TLR7 facilitates the response in mice versus TLR7/8 in humans. In fact, a study in TLR7-knockout mice demonstrated no siRNA-related response (25). Microarray analysis demonstrated that >400 genes were significantly altered in PBMCs in response to double- or single-stranded siRNAs (26).

Two classes of single-stranded TLR7/8 and TLR8 RNA agonist classes were identified that demonstrated species specificity, diverse target cells, and immune response profiles. These specific TLR8 RNA configurations communicate *via* TLR8 but do not induce IFN-α from TLR7-expressing plasmacytoid dendritic cells (PDCs); however, they induce Th1-like pro-inflammatory cytokine secretion by TLR8-expressing monocytes and myeloid dendritic cells (DCs). By contrast, RNA sequences containing the TLR7/8 motif communicate *via* TLR7 and TLR8 and induce cytokine secretion by TLR7- and TLR8-positive immunocytes. TLR8-specific RNA sequences trigger cytokine responses from bovine and human but not mouse, porcine, or rat immune cells, suggesting that the latter species lack the ability to properly respond to TLR8 RNA ligands. Thus, RNA sequences that induce specific TLR8-dependent immune responses must still be identified (27).

Some siRNAs use sequence-specific methods to act *via* TLR7/8 pathways and activate IFN-β. Immunostimulatory motifs, such as 5′-UGUGU-3′, 5′-GUCCUUCAA-3′, AU, UGGC, and GU, have been described on the siRNA sense strand (25, 27–33). Uridine/adenosine and uridine/guanosine nucleotides within the immunostimulatory RNA (isRNA) enable sensors for TLR8 and TLR7, respectively (12). Blunt-ended synthetic siRNAs use RIG-I to induce IFIT1 gene activation (34). TLR7/8-dependent cytokine production is triggered when uridine is present in RNA in a manner in which TLR7/8 agonist potency correlates with overall uridine moieties, while uridine replacement with adenosine abrogates the effect (31). Judge et al. reported that siRNAs created in non-viral delivery devices could be potent IFN and inflammatory cytokine inducers both *in vitro* in human blood and *in vivo* in mice. The formulated siRNA immunostimulatory activity and associated toxicities are nucleotide sequence-dependent. The immunostimulatory motifs 5′-UGU-3′ and 5′-UGUGU-3′ were identified (28). When examined in PDCs, the nine bases at the 3′ end (5′-GUCCUUCAA-3′) of the siRNA sense strand controlled its immunostimulatory activity, the immunostimulatory component of a strong IFN-α-inducing siRNA. Immunostimulation *via* siRNA was absent in mice lacking TLR7, which suggests that isRNA is identified in a TLR7-dependent and sequence-specific manner (25).

According to Sioud et al., human PBMCs recognize and respond to numerous single-stranded siRNA, both sense and antisense. isRNA motifs are usually more easily recognized *via* single-stranded siRNA than siRNA duplexes. The replacement of novel isRNA motifs with adenosines abrogated the immune activation. Interestingly, replacement of 2′-hydroxyl uridines with 2′-fluoro, 2′-*O*-methyl, or 2′-deoxy uridines blocked the immune activation. Thus, RNA immune recognition by TLRs can be prevented using 2′-ribose modifications of uridines (31).

The transfection of siRNAs causes IFN-mediated Jak–Stat pathway activation and global ISG upregulation. This effect is mediated through PKR protein kinase, which is dsRNA-dependent, activated by 21-bp siRNA sequences, and is required for siRNA-induced IFN-β upregulation. The RNAi mechanism is not dependent on the IFN system, as shown in cell lines lacking the specific components that mediate IFN action (13). The deletion of one nucleotide from the siRNA sequence prevented OAS1 induction, which suggests that its underlying mechanism involves a detector that can identify 19- but not 14-bp RNA constructs (35).

A summary of information related to the sequence-dependent stimulation of cellular pathways by siRNAs is given in Table 1.

**Table 1:** **Characteristics of immunostimulatory RNAs**.

Characteristic	Signaling pathway	Cytokines	Reference
5′-UGUGU-3′ motif	Toll-like receptor (TLR) 8	Interferon (IFN)-α	(27, 28)
5′-GUCCUUCAA-3′ motif	TLR7/8	IFN-α	(25)
GU or AU rich	TLR7/8	IFN-α, tumor necrosis factor (TNF)-α	(27, 28)
Uracil repeats	TLR7	IFN-α, interleukin-6, TNF-α	(30–33)
Blunt ended	Retinoic acid-inducible gene-I (RIG-I)	Type I IFN, p56	(34)
5′-Triphosphate	RIG-I	IFN-α, IFN-β	(36–38)
MicroRNA-like small interfering RNA	TLR7/8	IFN-α, TNF-α	(39)

*The sequence motifs and the corresponding stimulated cellular signaling pathways are given*.

## Vector-Associated Immune Stimulation

Bridge et al. showed that a frequently used shRNA sequence that includes plasmid and lentiviral systems can induce an IFN response and recommended IFN induction testing prior to declaring a target-specific effect. The use of the lowest effective shRNA vector dose is a simple way to limit the risk of stimulating an IFN response (14).

Pebernard and Iggo showed that shRNA-expressing lentiviral vectors with the U6 promoter more frequently exhibited ISG induction than lentiviral vectors using the H1 promoter. Further studies revealed that ISG induction was partially induced by an AA di-nucleotide near the transcription start site. A lamin shRNA was found to induce OAS1 when fused to VA RNA I, which can inhibit PKR, independent of U6 or H1 promoters (35). The use of lentiviral vectors to deliver shRNA of 21-mers or more to silence the plasminogen activator inhibitor-2 mRNA increased OAS1 expression and decreased the lentiviral vector titer needed to reduce shRNA expression and OAS1 induction without majorly impacting gene silencing efficacy (40).

*In vivo* studies have shown that peptide siRNA conjugates can induce unwanted immune responses. Also, siRNAs targeting p38 MAP kinase conjugated with penetratin, but not cholesterol or TAT (48–60), activated an innate immune response, likely *via* TLR3, TLR7, and/or TLR8 activation (31, 41). Significantly increased TNF-α and IL-12 p40 expressions and increased IFN-α release were observed after the intratracheal administration of penetratin–siRNA but not penetratin or siRNA alone, suggesting that penetratin-mediated siRNA delivery is accomplished differently from those of cholesterol and TAT conjugates or that the individual conjugates enter the cytosol and experience various biochemical outcomes (41).

## RNAi-Directed Immune Stimulation

Interestingly, in an RNAi study of WHV-infected PWHs, the RNAi-mediated WHV suppression enhanced inflammatory cytokine as well as MxA and MHC-I cellular gene expressions. The increased MHC-I and MxA expressions were identified in WHV-infected but not naïve PWHs, excluding the sequence-dependent immune stimulation of the siRNAs. In addition, MHC-I and MxA expression upregulation was observed when the siRNAs effectively degraded WHV RNAs, and the more effective the siRNAs, the higher the MxA and MHC-I induction levels. These data suggested that specific siRNAs inhibit the replication of hepadnaviruses but induce the cellular gene expressions that antiviral activity requires (16).

Further studies have shown that specific siRNAs targeting WHV as well as host genes such as mouse β-actin and GAPDH can upregulate the mRNA expressions of MxA, IFN-β, and IP-10 as well as reduce targeted mRNA expression. The enhanced ISG and inflammatory cytokine expressions were enhanced by viral and host gene silencing; an RNAi inhibitor disrupted ISG upregulation, but the lack of RNAi targets resulted in no ISG induction. Thus, immune stimulation under these circumstances must be mediated *via* RNAi or related factors and most likely by cleaved components resulting from the RNAi process. The RNAi-mediated immune stimulation was abolished using inhibitors for TLR3/7/8 and PKR signaling pathways but not RIG-I/MDA5 interference (17).

Collectively, these findings demonstrate that RNAi triggers immune activation *via* the TLR3/7/8 and PKR cascades. The so-called GU-rich non-self-RNA molecules compose RNAi-generated small viral RNA cleaved components that can upregulate antiviral RNAi *via* PKR and TLR3/7/8 signaling (Figure 1) (17).

## Overcoming the Challenges of RNAi-Associated Innate Immunity Stimulation

Because RNAi-associated immune stimulation reflects special siRNA sequences, siRNA delivery vehicle types, and RNAi-directed RNA cleavage products, it is possible to create strategies of avoiding RNAi-associated immune activation. First, known immunostimulatory motifs, such as 5′-UGU-3′, 5′-UGUGU-3′ (28), and 5′-GUCCUUCAA-3′ (25), should be lacking from the non-stimulatory siRNA sequences. Second, fewer immunostimulatory vectors should be selected for siRNA delivery. Third, a reduction in the number of siRNAs can attenuate RNAi-directed immune activation.

### Designing Non-Stimulatory siRNAs

#### Modification of siRNAs

Using the appropriate modification may enable siRNA-associated immune activation evasion without reducing RNAi potency. Base modifications can reduce immune activation, and the addition of modified nucleotides into siRNA suppresses unwanted immunostimulation. Morrissey et al. reported that the use of various 2′-modified nucleotides, including DNA bases, 2′-*O*-methyl purines, 2′-fluoropyrimidines, PS linkage modifications, and terminal inverted-dT bases at certain points could prevent siRNA immune activation (42). The use of 2-thiouracil or pseudouracil prevents immunostimulation mediated by RIG-I, reflecting the 5′-triphosphate (43), while 5-methyl-C and N6-methyl-A pseudouridine prevent RNA recognition by TLR3, TLR7, and TLR8 (44). Interestingly, RNA with 2′-ribose modifications, 2′-*O*-methyl in particular, avoided immune activation and suppressed the trans configuration-induced TLR signaling *via* isRNAs. The 2′-*O*-methyl modifications on siRNAs avoid TLR-mediated *in vivo* and *in vitro* hepatic immune system activation (45). The addition of 2′-*O*-Me guanosine or uridine into the siRNA sense strands creates non-inflammatory siRNAs without interfering ability loss but can selectively protect the vulnerable 5′ end of the guide strand from the effects of human serum-derived exonucleases (46).

The chemical influences preventing immune system activation must be carefully implemented to ensure siRNA-silencing activity. The replacement of uridine bases only with 2′-fluoro-, 2′-deoxy-, or 2′-*O*-methyl-modified counterparts can abrogate TLR siRNA immune recognition without reducing the potent siRNA-silencing activity (31, 47). Also, 2′-uridine-modified ssRNAs did not activate innate human immunity within blood cells (31), and the exchange of uridines and thymidines in the siRNA sense strand did not prevent siRNA silencing but inhibited innate immunity activation (48). The 2′-*O*-Me siRNAs targeting apolipoprotein B (apoB) and containing <20% modified nucleosides can facilitate potent target mRNA silencing and significantly decrease serum apoB and cholesterol levels (47).

Although 2′-modified RNA strands can prevent immune activation, those that are naturally modified might be unrecognized by TLR7/8 (49). The inhibition of endosome acidification *via* chloroquine and bafilomycin A1 can block the TLR3-, TLR7-, and TLR8-directed immunological activities of siRNAs without impacting the effect of RNAi (24, 50).

Studies in human monocytes have shown that even low 2′-*O*-methyl-modified RNA concentrations can prevent isRNA-induced TLR7 activation (9, 50). Chemically enhanced RNA can provoke isRNA and induce production of the immunosuppressive enzyme indoleamine 2,3-dioxygenase (IDO) (51). Robbins et al. reported that 2′-modified isRNAs antagonize TLR7/8 by abrogating isRNA-induced TLR signaling or loxoribine in both human and murine cells (52). Suppressive 2′-modified RNAs may comprise a new agent class for treating TLR8 and TLR7 signaling-triggered autoimmunity. Since TLRs are not bound by thymidine-modified siRNAs and 2′-deoxyuridines, such a change should be able to preserve TLR function and prevent immune activation (50).

## Enhancing RNAi Immune Activity

### Potential Benefits of Immunostimulatory siRNA (isiRNA) Use

Undesired activity of immunosuppressive cytokines and other negative regulators are expected to negatively impact immunity against tumors and virus-infected cells (53). Therefore, stimulators of immune and/or virus-infected cells that produce IFN and/or Th1 cytokines and can prevent cancer and viral-specific immune tolerance and restore immune surveillance in cases of cancer and viral infections are needed.

Stimulation with isRNA can induce the maturation of DCs and subsequent cytokine secretion, including IL-6 and IL-12, both of which are essential to CD4+ effector T cell- and Th1-type responses (24). Hyperactive bifunctional siRNAs targeting IDO, a key factor involved in immune suppression, can abrogate its genetic expression in human DCs and monocytes. Interestingly, when transfected with siRNA bearing 5′-triphosphate, immature monocyte-derived DCs activate T cells, showing that the DCs were mature enough to initiate T-cell activation, even without external stimuli. Taken together, these findings demonstrate that RNAi technology can regulate T-cell growth and development processes (50). Patients with cancer who were vaccinated with DC vaccines lacking IDO demonstrated this kind of clinical response (54).

### Rational Design of isiRNAs

#### Short RNA Structural and Sequence Requirements for Activating TLR7/8

The ability of short RNAs to sense TLR7/8 is uridine-dependent, and the residue positions of uridine within the secondary ssRNA structure could affect immunostimulation (7). As uridine levels increased, IFN-α and TNF-α were induced. At the ssRNA dose of 90 nmol/L, immunostimulation required four or more uridines. However, the use of eight or more uridines did not further increase the cytokine production. Collectively, these results suggest that the uridine content and secondary structure are involved in TLR7/8 sensing of ssRNAs (39). The combination of the immunostimulatory sequence (5′-UGUGU-3′) and western blot analysis of NP-specific siRNA revealed a strong antiviral effect of the isiRNA *via* a reduction in the number of mRNA copies (99.58%), virus-associated cell apoptosis, and nucleocapsid protein inhibition. The use of isiRNA was more effective than non-tagged siRNA, while the higher antiviral response of isiRNA reflected the upregulation of TLR7, MyD88, IRF7, and IFN-α (55).

#### miRNA-Like siRNA

Gantier et al. observed that the addition of anon-pairing uridine bulge similar to miRNA in the passenger strand of siRNAs greatly increased human immune cell immunostimulatory activity. In immunostimulatory assays, a comparison of the EGFP siRNA with or without bulge modification in immunostimulatory assays revealed greatly increased TNF-α induction (>10-fold at 500 and 750 nmol/L) and significantly greater IFN-α (at 500 nmol/L) induction. This bulge did not affect RNAi triggering of the EGFP siRNA in stable EGFP-expressing HEK 293T cells. The human papilloma virus 16 E6/E7 oncogene showed similar results. These results collectively demonstrate that different designs of the uridine bulge can greatly increase TNF-α and IFN-α expressions in human PBMCs independent of the siRNA sequence without preventing siRNA from entering the RNAi pathway (39). A protein array analysis of multiple cytokines confirmed the marked upregulations of TNF-α, IL-12 (p70), IL-1β, and IFN-γ and induced by the uridine bulge modification. G-CSF, GM-CSF, IL-4, IL-5, IL-7, IL-10, and IL-17 were significantly induced in two of three analyzed siRNAs. Due to the increased IFN-α production, the uridine modification of siRNA predictably provided significant protection against Semliki Forest virus infection compared to native siRNA, while the uridine bulge modification of 21-bp siRNA siβ-Gal significantly induced TNF-α and IFN-α in human PBMCs compared to the native variant (39). These results indicate that the uridine content increases, structural distortion, and bulge modification induced cytokine production. These data suggest that this combination of structural and sequence modifications specifically recruits human TLR8 versus TLR7 and may contribute to antiviral therapies (39).

#### 5′-Triphosphate-Modified siRNA

The addition of 5′-triphosphate (3p) to the siRNAs generated ligands for RIG-I, which subsequently became activated and induced type I IFN expression. The results showed that 3p-siRNAs (5′-end triphosphate siRNA) induced an antiviral type I IFN response that was dependent on RIG-I in HBV-infected primary human hepatocytes, HepG2.2.15 cells, and HBV transgenic mice (36–38). The 3p-siRNAs showed more pronounced and long-term suppression of HBV DNA replication and mRNA transcription than normal siRNAs targeting the same sequences, suggesting that 3p-siRNA might be a powerful antiviral molecule and potential therapeutic agent for treating chronic HBV infection (38).

Survivin, a new apoptosis inhibitor family member, is upregulated in human lung cancer and other malignancies. Studies in human lung cancer cells demonstrated that the 3p-siRNA targeting human survivin gene (3p-survivin-siRNA) induced a 3p-dependent type I IFN response and significantly downregulated lung cancer cell proliferation. In fact, its cancer-inhibiting effect exceeded that of conventional siRNA. Used with radiotherapy, 3p-survivin siRNA upregulates A549 cell cytotoxicity and increases the frequency of apoptosis (56). Using Bcl2-specific 3p-siRNA against melanoma, RIG-I signaling pathway activities mediated by 3p-siRNA combined with siRNA-mediated Bcl2 abrogation to induce massive metastasized lung tumor cell apoptosis. This effect *in vivo* required Bcl2 silencing, IFN, and natural killer cells as evidenced by rescuing with Bcl2 target mutation *via* Bcl2 mRNA site cleavage in lung metastases and Bcl2 protein downregulation in tumor cells (57).

**Figure 2:**
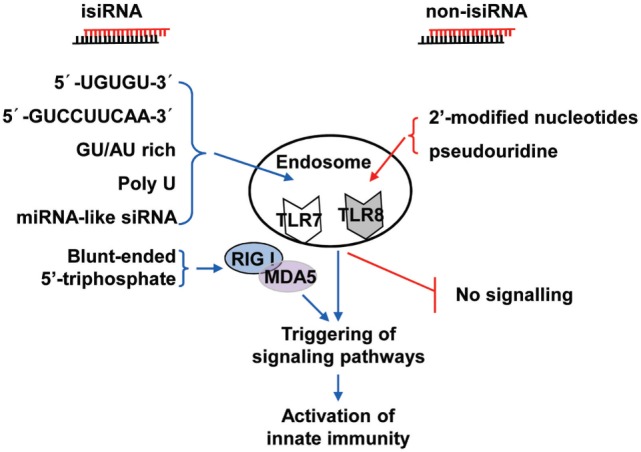
**Schematic representation of isiRNA and non-isiRNA**. RNA sequence requirements of isiRNA and non-isiRNAs were shown; isiRNA may trigger toll-like receptor (TLR) 7/8, or retinoic acid-inducible gene-I (RIG-I)/melanoma differentiation-associated protein 5 (MAD5) signaling pathways, leading to activation of innate immunity. isiRNA, immunostimulatory siRNA; non-isiRNA, non-stimulatory siRNA.

Collectively, these findings show that 3p-siRNAs can act as powerful bifunctional molecules with RIG-I activation and targeting knockdown and the potential to antagonize cancer and viral infections. isiRNAs enhance innate immunity levels and silence target genes in a sequence-specific manner, which gives RNAi a dual-action approach that directly targets knockdown and immunoenhancing activity. Thus, isiRNA may be tightly involved in antagonizing cancers and viral infections. RNA sequence requirements of isiRNA and rational design of non-stimulatory siRNAs were shown in Figure 2.

## Conclusion

The sequences of siRNA, the siRNA delivery vehicles, and the secondary RNAi products contribute to immune stimulation characterized by enhanced IFN and/or pro-inflammatory cytokine creation using the PKR, RIG-I, or TLR3/7/8 signaling pathways. Most of these immunostimulatory activities were considered off-target adverse effects; thus, non-stimulatory siRNAs are still needed. In other circumstances, immune stimulation is beneficial and isiRNAs with both RNAi and immunostimulatory activities may represent novel agents for treating cancers and viral infections.

## Author Contributions

ZM performed literature research, designed the review layout, and written the review. ML contributed to the conception of the review and revised the manuscript.

## Conflict of Interest Statement

The authors declare that the research was conducted in the absence of any commercial or financial relationships that could be construed as a potential conflict of interest.

## Footnotes

^1^https://clinicaltrials.gov.

^2^http://rnaimmuno.ibch.poznan.pl.
